# Beauvericin, a Bioactive Compound Produced by Fungi: A Short Review

**DOI:** 10.3390/molecules17032367

**Published:** 2012-02-24

**Authors:** Qinggui Wang, Lijian Xu

**Affiliations:** College of Agricultural Resource and Environment, Heilongjiang University, Harbin 150080, China

**Keywords:** beauvericin, bioactive compound, antibiotic, mycotoxin, *Fusarium* spp

## Abstract

Beauvericin is a cyclic hexadepsipeptide mycotoxin, which has insecticidal, antimicrobial, antiviral and cytotoxic activities. It is a potential agent for pesticides and medicines. This paper reviews the bioactivity, fermentation and biosynthesis of the fungal product beauvericin.

## 1. Introduction

**Figure 1:**
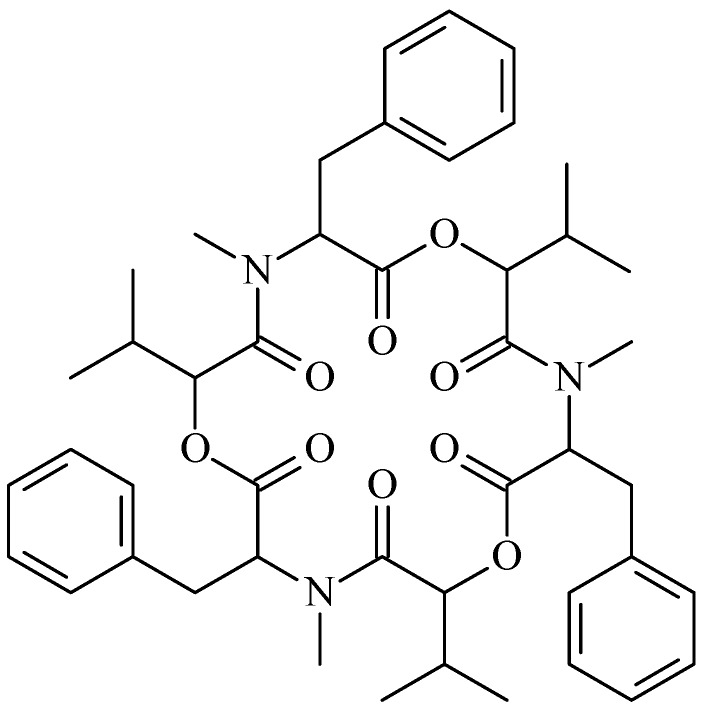
Beauvericin structure.

Beauvericin is a famous mycotoxin produced by many fungi, such as *Beaveria bassiana* and *Fusarium* spp. [1,2]. Beauvericin is a cyclic hexadepsipeptide (Figure 1) that belongs to the enniatin antibiotic family. It contains three D-hydroxyisovaleryl and three N-methylphenylalanyl residues in an alternating sequence [1,3]. It is structurally similar to the enniatins, which are also produced by a number of *Fusarium* species, but beauvericin differs in the nature of the N-methylamino acid. Owing to this difference between beauvericin and the enniatins, their bioactivities are obviously different [4]. Beauvericin was first isolated from *Beaveria bassiana*, which is a common and commercial entomopathogenic mycoinsecticide [1]. Beauvericin was one of the active constituents of *B. bassiana* [1,5,6,7] and was confirmed to have antimicrobial and anti-tumor activities. Because beauvericin is a mycotoxin, the toxicity to normal human cells has been investigated, and the detection methods for food safety have been developed; these studies are reviewed elsewhere [8,9,10,11,12,13,14]. However, there is no review about the biological activity and biosynthesis of beauvericin; therefore, this review is the first to focus on the bioactivity, fermentation and biosynthesis of beauvericin.

## 2. Bioactivity of Beauvericin

### 2.1. Insecticidal Activity

The insecticidal activity of beauvericin was first discovered by Hamill *et al.* [1]. Beauvericin was confirmed as the active compound from *B. bassiana *against *Artimia salina*, which was considered a model organism to study insecticidal activity. Subsequently, the insecticidal effect of beauvericin on a microgram level was investigated on *Calliphora erythrocephala*, *Aedes aegypti*, *Lygus* spp., *Spodoptera frugiperda* and *Schizaphis graminum* [3,15,16,17]. Although beauvericin has a strong insecticidal activity against a broad spectrum of insect pests, it has been applied as a commercial insecticidal agent for two main reasons: first, because of the movement of insects, using an entomopathogenic fungus produced beauvericin as an insecticidal agent has more advantages than the direct use of the compound. The entomopathogenic fungus could propagate in insect bodies and be spread widely by insect movement. The entomopathogenic fungus would give rise to a good control efficiency of insects even if a small amount of the spores of the entomopathogenic fungus were used. Second, the careful evaluation of beauvericin production should ensure that beauverin production does not increase above threshold limits set by the EPA [17]. Although beauvericin is not applied directly as a commercial insecticidal agent, the insecticidal mechanism of beauvericin is still worth investigating. There are few reports about the insecticidal mechanism of beauvericin. Despite similarities between the chemical structures of beauvericin and other cyclic hexadepsipeptide mycotoxins, beauvericin is more effective against *Aedes aegypti* [3] and may have a unique mechanism of action. The discovery of the active mechanism of beauvericin against insects will be helpful to find new commercial insecticidal agents, reduce the threat of insecticidal agents to human cells and reveal the active mechanism of other mycotoxins.

### 2.2. Antitumor Activity

**Figure 2:**
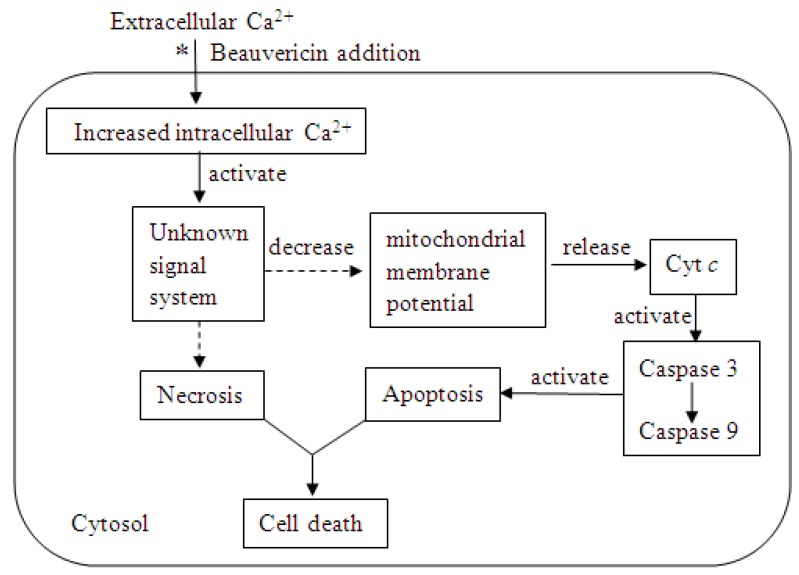
Mechanism of beauvericin cytotoxicity to a human leukemia cell (*beauvericin induced extracellular Ca^2+^ movement into the cell resulting in an increase in the level of intracellular Ca^2+^; dashed arrows indicate the specific mechanisms that are unclear).

Recently, more attention has been paid to the antitumor activity of beauvericin (Table 1) [18,19,20,21,22,23,24,25]. The cytotoxicity of beauvericin to human leukemia cells has been frequently reported. We summarize the partial mechanism in Figure 2 [20,21,22,23]. 

**Table 1:** The cytotoxicity of beauvericin.

Cell line	IC_50_ (μg/mL)	Reference
African green monkey kidney fibroblast Vero	10	[19]
Human monocytic lymphoma cells U-937	24	[21]
Human breast cancer BC-1	15	[19]
Human breast cancer MCF-7	1.4	[24]
Human CNS cancer (glioma) SF-268	1.8	[24]
Human epidermoid carcinoma KB	>20	[19]
Human leukemia cell CCRF-CEM	1–2	[20]
Human non-small cell lung cancer (NSCLC) A549	2.4–7.8	[22]
Human non-small cell lung cancer NCI-H460	1.1	[24]
Human pancreatic carcinoma MIA Pa Ca-2	1.3	[24]
Human promyelocytic leukemia HL-60	12	[21]
Human retinoblastoma Y79	0.4–4	[25]

First, beauvericin induces movement of extracellular Ca^2+^ into the cytosol, which leads to an increased intracellular Ca^2+^ level. The “unknown signal system” is activated by a high level of Ca^2+^ and results in the release of Cyt *c* from the mitochondria. Finally, the caspase that is activated by Cyt *c* triggers apoptosis. To date, it is unclear how an increased intracellular Ca^2+^ concentration regulates the signal system resulting in cell death. Reports on the cytotoxicity of beauvericin to other cell lines also found that its mechanism is related to Ca^2+^ flux [18,26,27,28]. Thus, the unknown part shown in Figure 2 needs further investigation. The cytotoxic mechanism of beauvericin for leukemia cells could be used to determine other mechanisms of beauvericin cytotoxicities, including its role as an insecticide and an antifungal compound.

### 2.3. Antimicrobial Activity

#### 2.3.1. Antibacterial Activity

Beauvericin has a strong antibacterial activity against human, animal and plant pathogenic bacteria (Table 2) [29,30,31,32], with no selectively between Gram-positive and Gram-negative bacteria. Unlike other antibiotics (such as penicillin) that block the peptidoglycan biosynthesis of Gram-positive bacteria, the bacterial cell wall is not the antibacterial mode of action of beauvericin, although these antibiotics and beauvericin are both from amino acids that are produced by fungi. It is possible that other cell organelles or enzyme systems are the targets of beauvericin. Despite its broad-spectrum antibacterial activity, the antifungal activity of beauvericin as a single agent is rarely reported. Therefore, the target of beauvericin is different between bacteria and fungi and could include targets such as the ribosome or the cell nucleus. The activity of beauvericin should be investigated against drug resistant bacteria. Based on the antibacterial activity against plant pathogens [32], beauvericin could be utilized in the control of non-food crop diseases. As a potential antibacterial agent, beauvericin could be used to solve the problems of drug resistance, deadly bacterial infections [30,31] and non-food crop disease.

**Table 2:** Bacterial strains that are inhibited by beauvericin.

Gram-positive bacteria	Reference	Gram-negative bacteria	Reference
*Bacillus *spp.	[29,32]	*Agrobacterium tumefaciens*	[32]
*Bifidobacterium adolescentis*	[29]	*Escherichia coli *CECT 4782	[31]
*Clostridium perfringens*	[29,31]	*Escherichia coli*	[32]
*Enterococcus faecium*	[31]	*Pseudomonas aeruginosa*	[31]
*Eubaterium biforme*	[29]	*Pseudomonas lachrymans*	[32]
*Listeria monocytogenes*	[31]	*Salmonella enterica*	[31]
*Mycobacterium tuberculosis*	[30]	*Shigella dysenteriae*	[31]
*Peptostreptococcus* spp.	[29]	*Xanthomonas vesicatoria*	[32]
*Staphylococcu haemolyticus*	[32]	*Yersinia enterocolitica*	[31]
*Paenibacillus* spp.	[29]		

#### 2.3.2. Antifungal Activity

The lack of antifungal activity of beauvericin as a single agent could be because it is a fungal product. However, Zhang* et al.* [33] and Fukuda *et al.* [34] report the antifungal activity of beauvericin in combination with ketoconazole or miconazole. Beauvericin (0.5 mg/kg) combined with ketoconazole (0.5 mg/kg) had remarkable antifungal activity against *Candida parapsilosis*, which can quickly cause high mortality rates, particularly in neonates. Both beauvericin and ketoconazole alone have little to no effect on *C. parapsilosis*. If the antifungal mechanism of beauvericin is similar to the cytotoxic mechanism in leukemia cells, it would indicate that the fungus itself can inhibit the “unknown signal system” (Figure 2) until another compound, such as ketoconazole, is added to unlock the signaling system. The method of combining beauvericin with another compound offers a new way to develop and utilize the biological activity of beauvericin.

#### 2.3.3. Antiviral Activity

The antiviral activity (IC_50_ 1.9 μM) [4] of beauvericin has also been detected. According to Shin *et al.* [4], beauvericin is the most effective inhibitor of the cyclic hexadepsipeptides that inhibit HIV-1 integrase. Enniatins have a comparatively weak activity despite having a similar structure, which implies that the activity of beauvericin could be due to the primary structural difference, N-methylation. Viral infections can result in fatal and epidemic diseases. Therefore, the antiviral activity of beauvericin should be investigated for its potential clinical effects and activity against other serious viruses, such as HBV, SARS, H1N1 and AIV. 

## 3. Biosynthesis of Beauvericin

**Figure 3:**
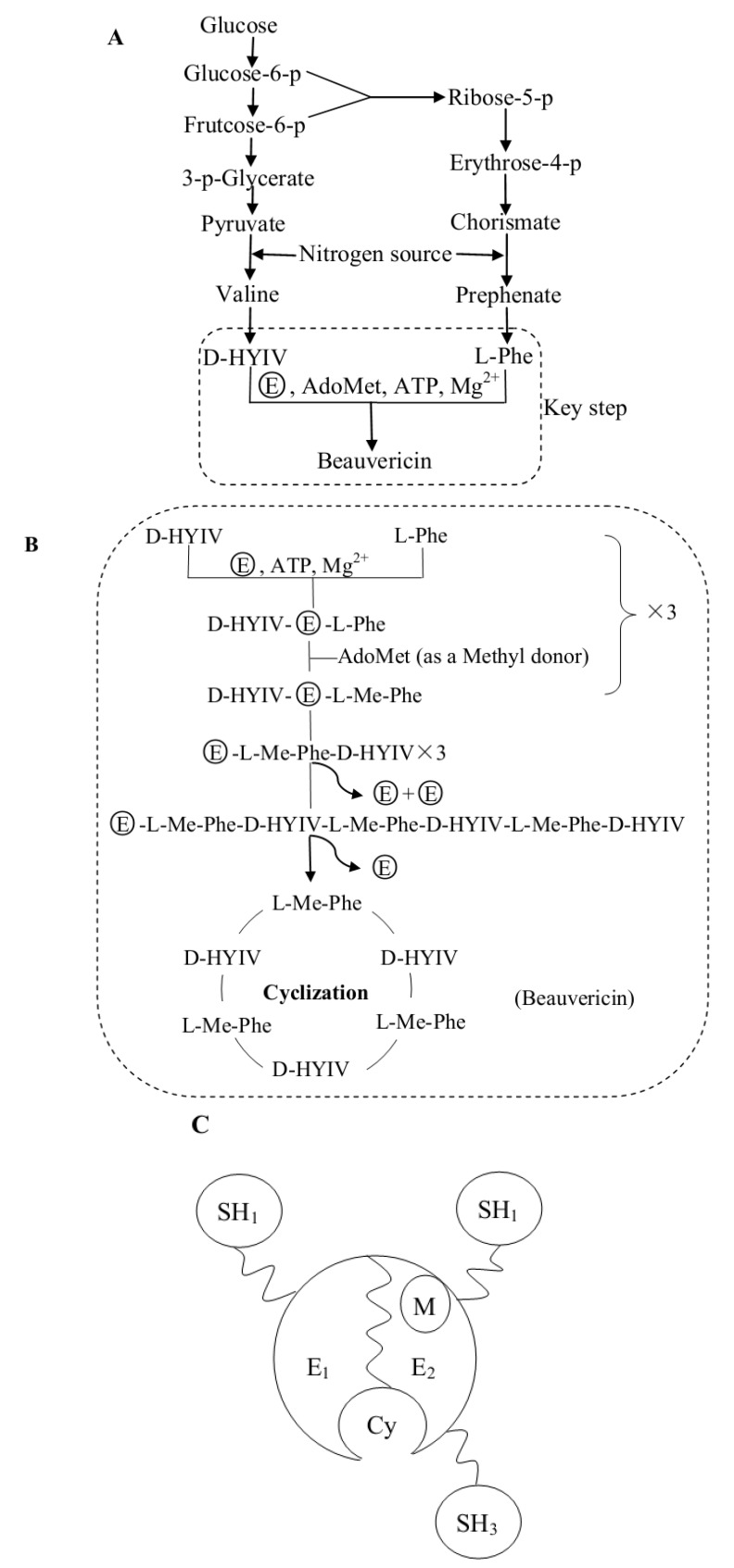
**A**, Beauvericin biosynthesis pathway; **B**, The specific substeps of the key step (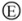 represents the beauvericin synthetase; the dashed frame indicates the key step of beauvericin synthesis); **C**, The possible structure of the beauvericin synthetase (E_1_ is the D-HYIV module; E_2_ is the L-Phe module; SH_1,2,3_ are the 4'-phosphopantetheine residues corresponding to D-HYIV, L-Phe, and the linear hexadepsipeptide acceptor; M is the N-methyltransferase domain; Cy is the cyclization cavity).

Based on the biosynthetic studies of beauvericin, in addition to a comparison to cyclic depsipeptide biosynthesis, we built a beauvericin biosynthetic pathway as shown in Figure 3A, B, C [35,36,37,38,39,40]. In Figure 3A, the “nitrogen source” could be any amino acid that offers nitrogen to L-phenylalanine or valine by transamination. Also, a hexose or a pentose could be the carbon source instead of glucose. Of seven potential carbon sources, glucose was the most effective for beauvericin biosynthesis, as reported by Xu *et al.* [41]. The last step in the pathway is key (Figure 3A) and is strictly dependent on the presence of the amino acid L-phenylalanine (L-Phe), the hydroxy acid D-hydroxyisovaleric acid (D-HYIV), ATP/Mg^2+^, S-adenosyl-methionine (AdoMet), and the beauvericin synthetase. AdoMet is the source of the methyl group for the L-phenylalanyl residues. The beauvericin synthetase (shown in Figure 3C) is a multifunctional enzyme that catalyzes depsipetide formation and is a single polypeptide chain with a molecular mass of approximately 250 kDa. Beauvericin biosynthesis is catalyzed by the beauvericin synthetase via a nonribosomal, thiol-templated mechanism. As shown in Figure 3B, we summarize the five intermediates in the key step of beauvericin biosynthesis. First, two beauvericin synthetase modules (Figure 3C) are activated by the corresponding L-Phe and D-HYIV, which are covalently attached to the enzyme-bound 4'-phosphopantetheinyl arm as thioesters. Next, the L-phenylalanyl residues are *N*-methylated by AdoMet. Third, a peptide bond is formed. Fourth, a linear hexadepsipeptide intermediate is synthesized. In the final step, the linear hexadepsipeptide is cyclized to make beauvericin. The optimum pH for beauvericin formation was pH 7.2, and the optimum temperature was 25~27 °C; the beauvericin synthetase was inactive if the temperature was more than 30 °C [35,37]. To date, the amino acid sequence and the gene encoding the beauvericin synthetase are unclear. However, the specific recognition sites of the beauvericin synthetase vary considerably and may contribute to the biosynthesis of beauvericin analogs. For example, the L-Phe could be replaced by a different aromatic or aliphatic amino acid [37,38]. The analogs could give more support to apply beauvericin as a lead for potential medicines and pesticides.

## 4. Production of Beauvericin by Fungal Fermentation

At present, many compounds that were isolated from fungi are used as medicines and pesticides. Fermentation conditions for beauvericin production have been studied recently as it is a potential commercial fungal product. The conclusions from carbon and nitrogen source screening for beauvericin production indicated that the optimal carbon source was glucose, the optimal organic nitrogen source was peptone and the optimal inorganic nitrogen source was NaNO_3_ [40,41]. Using valine and phenylalanine as nitrogen sources did not increase beauvericin production, which indicated that the fungus offered enough precursors for beauvericin production by itself when an optimal medium was used. Substrate (glucose) inhibition was identified in the fermentation of beauvericin using *F. redolens* Dzf2. The fed-batch (glucose was fed to the culture) was successfully used in beauvericin fermentation, which provided ample substrates for beauvericin production and reduced the substrate inhibition [42]. Beauvericin is an intracellular product; only a small amount of beauvericin is exported to the medium. Interestingly, macroporous polystyrene can efficiently extract beauvericin from mycelium cells. An integrated fermentation, with an *in situ* product recovery process using macroporous polystyrene, was used to effectively enhance beauvericin recovery in the mycelial liquid culture [43]. It facilitated the extraction and separation of beauvericin from the fungal mycelia. In addition, a modified Monod model of the fed-batch method for efficient beauvericin production was developed. This model with substrate (glucose) inhibition, together with the stoichiometric relationships for biomass, substrate and product, can be applied to predict the optimization scheme for fed-batch fermentation. Overall, mycelial fermentation of fungi, such as *Fusarium* spp., is a feasible and promising process for the production of beauvericin. Although beauvericin production was increased to 400 mg/L in the mycelial liquid culture by optimization of the fermentation process [43], the quantities of beauvericin produced were lower than those of other industrial and commercial antibiotics. The study of the fermentation process and the biosynthesis should be continued to improve beauvericin production. For example, two-phase fermentation could be investigated to increase beauvericin production. It is possible that intracellular beauvericin could be extracted by using an organic and non-polar solution in the fermentation process to enhance production.

## 5. Perspectives

The detection of beauvericin has been adequately studied (picogram level) [8,9,10,11,12,13,14], but it is our contention that the bioactive development of beauvericin is relatively ignored. Studies on medicines and pesticides indicate that it will be impossible to find a “perfect compound” without any side effects in a long time. Therefore, we believe that development of existing compounds (such as beauvericin) could be the best way to discover new medicines and pesticides. It has been confirmed that beauvericin is a fungal product with not only various kinds of bioactivities, but also with unique uncharacterized active mechanisms. From now on, we should put our focus to unravel each active mechanism of beauvericin rather than only testing more microorganisms or cell lines. We should pay more attention to the control or reduction of the beauvericin risk to humans rather than setting up more detection methods for beauvericin with all kinds of instruments. For example, it is critical to investigate the reason why fungi cannot be inhibited by beauvericin alone. If this mechanism is revelaed, it will be expected that we can better manipulate the bioactivity of beauvericin, to some extent, similar to a “light switch” that we can control. It will be a great discovery in pharmacology and toxicology. Beauvericin is a bioactive compound with the potential for use as a medicine or a pesticide, especially because of its potential use in deadly diseases such as cancer or viral and bacterial infections. We believe beauvericin will become a star compound. In addition, beauvericin can only be biosynthesized by several specific fungal genera: *Beauveria*, *Paecilomyces*, *Polyporus*, *Isaria* and *Fusarium* [2,44,45]. It has been suggested that the content of beauvericin can serve as a chemotaxonomic marker of fungi. The reason why beauvericin is only biosynthesized by those specific fungal species should be investigated. According to the biosynthetic mechanism, fermentation process for beauvericin production will increase its commercial interest. Because of its broad and significant bioactivities, beauvericin could become a commercial product from fungi in the future.
